# Novel nonsense variants in *SLURP1* and *DSG1* cause palmoplantar keratoderma in Pakistani families

**DOI:** 10.1186/s12881-019-0872-1

**Published:** 2019-08-23

**Authors:** Abida Akbar, Claire Prince, Chloe Payne, James Fasham, Wasim Ahmad, Emma L. Baple, Andrew H. Crosby, Gaurav V. Harlalka, Asma Gul

**Affiliations:** 1 Department of Biological Sciences, International Islamic University, H-10, Islamabad, 44000 Pakistan; 2 College of Medicine and Health, RILD Wellcome Wolfson Centre, University of Exeter, Royal Devon & Exeter NHS Foundation Trust, Barrack Road, Exeter, EX2 5DW UK; 3 Department of Biochemistry, Faculty of Biological Sciences, Quaid-e-Azam University (QAU), Islamabad, Pakistan; 4 Rajarshi Shahu College of Pharmacy, Malvihir Buldana, Maharashtra, Post code 443001 India

**Keywords:** Mal de Meleda, Palmoplantar keratoderma, *SLURP1*, *DSG1*, Mutation, Variant, Exome sequencing

## Abstract

**Background:**

Inherited palmoplantar keratodermas (PPKs) are clinically and genetically heterogeneous and phenotypically diverse group of genodermatoses characterized by hyperkeratosis of the palms and soles. More than 20 genes have been reported to be associated with PPKs including desmoglein 1 (*DSG1*) a key molecular component for epidermal adhesion and differentiation. Mal de Meleda (MDM) is a rare inherited autosomal recessive genodermatosis characterized by transgrediens PPK, associated with mutations in the secreted LY6/PLAUR domain containing 1 (*SLURP1*) gene.

**Methods:**

This study describes clinical as well as genetic whole exome sequencing (WES) and di-deoxy sequencing investigations in two Pakistani families with a total of 12 individuals affected by PPK.

**Results:**

WES identified a novel homozygous nonsense variant in *SLURP1,* and a novel heterozygous nonsense variant in *DSG1,* as likely causes of the conditions in each family.

**Conclusions:**

This study expands knowledge regarding the molecular basis of PPK, providing important information to aid clinical management in families with PPK from Pakistan.

**Electronic supplementary material:**

The online version of this article (10.1186/s12881-019-0872-1) contains supplementary material, which is available to authorized users.

## Background

Palmoplantar keratoderma (PPK) is a heterogeneous entity of both genetics and acquired keratinization disorder, which is characterized by persistent marked epidermal thickening of palms and soles [1]. Hereditary PPKs comprising an increasing number of entities with different prognoses, which may be associate with cutaneous and extracutaneous manifestations [2].

Depending on different patterns of hyperkeratosis, PPKs are further classified into four distinct types: diffuse, striate, focal and punctate [3, 4]. So far, deleterious mutations in > 20 genes have been reported in pathogenesis of different forms of hereditary PPKs [3, 4]. In last few years, advent of cutting edge genetic techniques such as whole genome microarray scans and whole exome sequencing have incredibly accelerated the identification of disease causing variants in many genes involved in various inherited forms of PPKs, and thus significantly increasing understanding about intricate molecular mechanisms of heterogeneous disorders, consecutively aiding valuable genetic counselling and patient care [3].

Mal de Meleda (MDM), a type of transgradient palmoplantar keratoderma (PPK), is a rare autosomal recessive disorder. Luca Stulli, a Croatian born scientist in 1826 first described Mal de Meleda on the Adriatic Meleda island (now Mljet) [5]. The disease can feature other potentially disfiguring effects on the hands and feet that can severely impact function.

The disease onset is soon after birth and is clinically characterized by erythema, transgradients and progradients hyperkeratosis of palms and soles with well demarcated borders and hypohydrosis. Other associated features are brachydactyly, nail abnormalities and lichenoid plaques [6]. Rigorous keratoderma can lead to deformity in hands and feet and gradually this may results into severe impairment [7, 8].

Furthermore, previous reports have shown that MDM may be caused due to mutations in the *SLURP1* gene (previously known as ARS-B gene) encoding a secreted toxin-like mammalian lymphocyte antigen 6/urokinase-type plasminogen activator receptor-related protein 1(*SLURP1*). Expression of *SLURP1* is reported in epithelium, stomach, sensory nerve cells, gums, esophagus and immune cells with highest level in keratinocytes especially in palms and soles [9–11].

Striate PPK type I is a rare type of PPK and shows the autosomal dominant mode of inheritance associated with *DGS1* heterozygous mutation. Clinical features of this condition are linear hyperkeratotic lesions on the palms extending along the length of fingers and associated with thick patches of diffuse hyperkeratosis on the soles [12].

Heterozygous mutation in *DSG1* gene in an autosomal dominant pattern have also been reported in focal PPK in a Libyan family, and in a Jewish Yemenite family with diffuse PPK [13, 14], a discovery which elucidates that different patterns of palmoplantar involvement may result from mutations in the *DSG1* gene. Additionally, bi-allelic mutations in *DSG1* gene have also been recently reported in the severe SAM syndrome, characterized by sinusitis, palmoplantar keratoderma, erythroderma, multiple allergies and metabolic defects, with heterozygous mutation carriers only presenting hyperkeratotic palmoplantar lesions [15].

Here we report findings regarding investigations of two families from Pakistan with clinically-defined PPK, for which the specific genetic basis was unclear.

## Methods

### Genetic studies

The research work presented in this manuscript was approved by the Ethical Review Boards Committee at International Islamic University, Islamabad, Pakistan (IIUI; Pakistan). Informed written consent was obtained for all participants for the collection of blood samples, with clinical evaluations and family histories performed by a dermatologist. Extraction of high quality genomic DNA from the whole blood was carried out by using the ReliaPrep™ kit (Blood gDNA Miniprep System, Promega) following the manufacturer’s protocol. Whole exome sequencing (WES) was undertaken on a NextSeq500 (Illumina, CA, San Diego, USA) with targeting using Agilent Sure select Whole Exome v6. The reads were aligned using BWA-MEM (v0.7.12), with mate-pairs fixed and duplicates removed using Picard (v1.129). InDel realignment and base quality recalibration were performed using GATK (v3.4–46). SNVs and InDels were detected using GATK Haplotype Caller or SnpEff tool (http://snpeff.sourceforge.net/SnpEff_manual.html), and annotated using Alamut batch (v1.4.4). Read depth was determined for the whole exome using GATK Depth of Coverage.

Primer3 web software was used to design the allele-specific primers (primer sequences are available upon request) to validate and verify the segregation of identified variants via Sanger sequencing. Polymerase chain reaction (PCR) was performed for all affected and healthy individuals of recruited families by using allele-specific primers following standard conditions, with products sequenced by Source Bio-Science Life Sciences (https://www.sourcebioscience.com/).

## Results

### Subjects

**Fig. 1:**
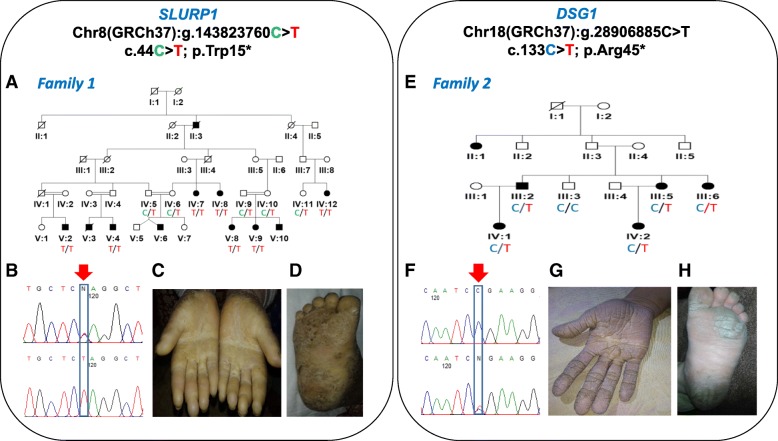
*SLURP1* Chr8(GRCh37):g.143823760C > T; c.44C > T; p.Trp15 (Family 1). *DSG1* Chr18(GRCh37):g.28906885C > T; c.133C > T; p.Arg45* (Family 2). **a**, **e** Simplified pedigrees of the extended Pakistani families investigated with genotypes of affected and unaffected ndividuals. **b** Electropherograms showing the DNA sequence at the position of *SLURP1* c.44 C > T in a heterozygous healthy carrier and homozygous affected individual. **c**-**d** Palmoplantar keratoderma in an individual homozygous for *SLURP1* c.44 C > T. **f** Electropherograms showing the DNA sequence at the position of *DSG1* c.133C > T in a wild type control and heterozygous affected individual. **g**-**h** Palmoplantar Keratoderma in an individual heterozygous for *DSG1* c.133C > T

Pedigree analysis was indicative of an autosomal recessive inheritance pattern of family 1, and an autosomal dominant mode of inheritance of family 2 (Fig. 1). All 12 living affected individuals with PPK as well as 6 unaffected (healthy) individuals including parents and siblings from both families (Family 1 and 2) were investigated. The seven affected individuals from family 1:IV:7, IV:8, IV:12, V:2, V:4, V:8 and V:9 were 27, 22, 45, 16, 11, 15 and 13 years of age respectively at the time of examination, while the five affected individuals from family 2: III:2, III:5, III:6, IV:1 and IV:2 were 28, 36, 40, 12 and 8 years of age respectively. On the basis of basic clinical dermatological examination, PPK was the main finding exhibit in all patients (affected members) of the recruited families.

Disease onset was from 3 months to 1 year. Affected individuals of family 1 show cuff-like pattern with well demarcated margins and waxy yellow tone on hands and feet. Diffuse hyperkeratosis of hand and feet was common in patients. Fingers were tapered towards the tips and flexion deformity due to contractures was observed in 2 (IV:7 and IV:8) patients. Knuckle pads were observed in interphalangeal joints and hyperhidrosis was also common in all patients. Patients in family 2 showed diffuse hyperkeratosis with cracks and fissuring of the volar surface of the digits of hands and soles, toes were observed in two siblings. All other patients have mild hyperkeratosis. Mild to severe deafness was observed in patients and one patient (III:5) was deaf as well as mute. Both families’ phenotypes are summarized in Table 1 and Fig. 1.

**Table 1:** Clinical phenotypes of study participants of family 1 and 2

	Family 1							Family 2				
Individuals	IV:7	IV:8	IV:12	V:2	V:4	V:8	V:9	III:2	III:5	III:6	IV:1	IV:2
Age	27 years	22 years	45 Years	16 years	11 years	15 years	13 years	28 years	36 years	40 years	12 years	8 years
Sex	F	F	F	M	M	F	F	M	F	F	F	F
Disease onset	1 year	1 year	6 months	6 months	1 year	3 months	6 months	By birth	By birth	By birth	By birth	By birth
Inheritance	Autosomal Recessive							Autosomal Dominant				
Diffuse PPK	+	+	+	+	+	+	+	+	+	+	+	+
Scale colour	Yellowish	Yellowish	Yellowish	Yellowish	Yellowish	Yellowish	Yellowish	–	–	–	–	–
Cuff like margins	++	++	++	++	++	++	++	–	–	–	–	–
Pseudoainhum	–	–	–	–	–	–	–	–	–	–	–	–
Cracked Hypekeratosis	–	–	–	–	–	–	–	++	+++	+	+	+
Deafness	–	–	–	–	–	–	–	Mild	Complete	Mild	Mild	Mild
Speech abnormality	–	–	–	–	–	–	–	Mild	Complete	–	–	–
Diffuse hyperkeratosis	–	–	–	–	–	–	–	Severe	Severe	Mild	Mild	Mild
Teeth, hairs and nails	Normal	Normal	Normal	Normal	Normal	Normal	Normal	Normal	Normal	Normal	Normal	Normal
Finger deformity	–	++	++	+	–	–	–	–	+	+	–	–
Hyperhidrosis	+	+	+	+	+	+	+	–	–	–	–	–
Cardiomyopathy	–	–	–	–	–	–	–	–	–	–	–	–

+ = presence of feature, − -absence of feature, +++ = present in severe form

All patients of family 1 and 2 were intellectually normal and hair, nail, teeth and cardiac anomalies were not observed in any of the patient. Disease conditions worsened due to aging.

### Genetic findings

To identify the causative gene mutation, single affected individual from each of the family was initially selected to perform WES (subject IV:12 of family 1 and III:2 of family 2, Fig. 1) to generate a profile of rare and novel sequence variants, with regard to mode of inheritance in each of the families. After that, exome data was first reviewed to identify pathogenic variants in disease associated genes, filtering for highly likely deleterious (nonsense, frame-shift, non-synonymous exonic or splice-site) variants for comparison with allele frequencies in online genome databases (including the Exome Aggregation Consortium; ExAC, the 1000 Genomes Project and the Genome Aggregation Database; gnomAD). This identified a single candidate novel homozygous nonsense variant [NM_020427.2:c.44G > A; Chr8:143823760C > T (GRCh37)] in the first coding exon of *SLURP1* gene (Fig. 1) in family 1. This variant leads to substitution of tryptophan by a premature termination codon and is at the evolutionary conserved position 15 (p.Trp15*). In family 2, a heterozygous variant [NM_001942.3:c.133C > T; Chr18:28906885C > T (GRCh37)] was identified in coding exon 3 of the *DSG1* gene (Fig. 1), which is predicted to result in a premature stop codon (p.Arg45*). The *SLURP1* gene variant in family 1 is not listed in online genome databases and segregates as predicted for an autosomal recessive form in family 1. The *DSG1* gene variant identified of family 2 is listed in the gnomAD browser database in 1 Latino individual in heterozygous form out of 31,370 genomes, corresponding to a minor allele frequency of 0.00003188; both variants are summarized in Additional file 1: Tables S1 and S2 alongside all other reported disease-associated *SLURP1* and *DSG1* variants.

## Discussion

*SLURP1* has been localized to the granular layer of epidermis [16], where it functions as part of nicotinic acetylcholine receptors found on keratinocyte cells as a pro-apoptotic protein [17]. Arredondo et al. [17] demonstrated that keratinocytes are stimulated by SLURP1 through nicotinic acetylcholine receptor, leading to decline in keratinocytes cell number, indicative of the inhibitory and regulatory nature of SLURP1. Therefore, when SLURP1 is non-functional, as seen in Mal de Meleda, severe hyperkeratosis results due to improper keratinocyte apoptosis regulation [8, 18].

We identified nonsense variant in family 1 which causes substitution of evolutionarily conserved tryptophan at 15th amino acid position in *SLURP1* by a premature termination codon. Nonsense variant (c.129C > A; p.Cys43*) is also reported in exon 2 of *SLURP1* gene in a Turkish family. Similarly another nonsense mutation (c.286C > T; p.Arg96*) is also found in exon 3 in Croatian family and is predicted to truncate protein synthesis via nonsense-mediated mRNA decay [19, 20]. Family reported in this study have same clinical features to previously reported Mal de Meleda families.

The *SLURP1* gene mutation p.Gly86Arg is most often found in sporadic patients with MDM of Asian origin [21, 22].

So far 20 mutations in *SLURP1* are reported to cause Mal de Meleda, a form of PPK (Additional file 1: Table S1,). c.44G > A; p.Trp15Term is the second variant identified in Pakistan apart from c.2 T > C, p.Met1Thr variant which was recently reported [23].

“Desmoglein” comprises of the two Greek words “desmos” for “tie” and “glein” for “glue-like.” Perturbations of desmoglein expression in the epidermis have been known to impact cell adhesion properties. *DSG1* is distinctively located, just above the stratum germinativum, to be candidate of epidermis stratification and differentiation [24]. A study in which *DSG1* was down regulated in skin culture confirmed the importance of *DSG1* for directing those functions [25].

In all reported PPKs cases where *DSG1* gene variants (frameshift or nonsense) have been reported, there is evidence that affected protein haploinsufficiency leads to the striate, focal PPK and striate PPK with wooly hair and cardiomyopathy. Haploinsufficiency is predicted to cause through nonsense mediated mRNA decay because of premature termination codons [26, 27]**.** Interestingly, a heterozygous *DSG1* mutation has also been reported in focal PPK [13].

To date, 31 mutations (8 nonsense mutations, 14 frame-shift variants and 9 splice-site variants) in *DSG1* have been reported to cause striate/focal PPK (Additional file 1: Table S2). In 2009, Dua-Awereh et al. reported five heterozygous variants (p.Arg26*; c.373-2A > G; c.515C > T; c.1266-3C > G and c.1399delA) in *DSG1* gene in five families with autosomal dominant striate PPK [28]. Thus, c.133C > T; p.Arg45* variant identified in this study is the sixth mutation underlying dominantly inherited form of striate PPK in Pakistan.

MDM presented a consistently severe phenotype than Nagashima form of PPK. MDM shows progressive hyperkeratosis among all PPKs and causes flexion contracture and constricting band [29]. While, Nagashima PPK is characterized by non-progressive and mild hyperkeratosis and does not show flexion contracture and constricting band [30, 31]**.** Nagashima PPK is caused by biallelic loss of function mutation in *SERPINB7* while, MDM is caused by *SLURP1* gene mutation [20]. Therefore, MDM is genetically distinct from Nagashima PPK [32]**.** PPKs are diagnosed on the basis of differential diagnosis to find out the disease entity. Differential diagnosis of PPK is summarized in Table 2.

**Table 2:** Differential diagnosis of PPKs

Name	Disease Type	Clinical Features	Histopathology	Gene
Mal de Meleda	**Diffuse PPK**	1. Soon after birth 2.Severe diffuse yellow and waxy thick hyperkeratosis in a ‘glove-and-socks’ distribution 3.Sharp demarcation 4. Autosomal Recessive	1.Nonepidermolytic pattern 2.Increased stratum lucidum 3.Prominent perivascular inflammatory infiltrate	*SLURP1*
Unna-Thost		1.Soon after birth to early childhood 2.Diffuse yellowish thick hyperkeratosis with sharp demarcation at the volar border 3. Autosomal Dominant	1.Epidermolytic pattern (perinuclear vacuolization and granular degeneration of keratinocytes in the spinous and granular layer)	*KRT1, KRT9*
Greither Disease		1.Soon after birth to childhood/adolescence 2. Diffuse red/yellow moderate to severe hyperkeratosis 3. Autosomal Dominant	1.Epidermolytic pattern	*KRT1*
Nagashima PPK		1.Mostly within infancy 2. Diffuse mild reddish hyperkeratosis, red rim; white spongy appearance after water exposure 3. Autosomal Dominant	1.Nonepidermolytic pattern	*SERPINB7*
Striate PPK				
Striate Type I PPK	**Focal PPK**	1.Childhood to adolescence 2. Linear hyperkeratotic distribution on palms and palmar surface of the fingers 2.Focal hyperkeratosis at trauma-prone sites on soles 3.Autosomal Dominant	1.Hyperkeratosis 2. Widening of intercellular spaces in the spinous and granular layer	*DSG1*
Striate Type II PPK		1.Childhood to early adulthood 2. Linear hyperkeratotic distribution on palms and palmar aspect of fingers 3.Focal hyperkeratosis at trauma-prone sites on soles 4. Autosomal Dominant	1.Hyperkeratosis 2.Widening of intercellular paces and condensation of the keratin filament network in suprabasal cell layers	*DSP*
Punctate PPK				
Punctate PPK Type IA	**Focal PPK**	1.Late childhood to adulthood 2.Multiple hyperkeratotic papules with central indentation 3.Worsening of papules upon exposure to water 4. Autosomal Dominant	1.Hyperkeratosis and hypergranulosis with central epidermal depression	*AAGAB*
Punctate PPK Type IB		1.Late childhood to adulthood 2.Multiple hyperkeratotic papules with central indentation 3. Autosomal Dominant	1.Hyperkeratosis and hypergranulosis with central epidermal depression	*COL14A1*
Punctate PPK Type II		1.Puberty to early adulthood 2.Multiple spiny keratosispits with keratotic plugs (late onset) 4. Autosomal Dominant	1. Columns of parakeratotic corneocytes (cornoid lamellae) 2.Superficial epidermal depression where the granular layer is reduced or absent	Unknown
Punctate PPK Type III		1.Adolescence to adulthood 2.Translucent hyperkeratotic papules, sometimes umbilicated, on lateral aspects of palms and soles 3. Autosomal Dominant	1.Hyperkeratosis and hypergranulosis 2.Decreased number of fragmented elastic fibres	Unknown

*PPK*, Palmoplantar keratoderma; *SLURP1*, Secreted lymphocyte antigen 6 (LY6)/urokinase-type plasminogen activator receptor (uPAR)-related protein-1; *KRT*, Keratin;*SERPIN7*, serpin peptidase inhibitor, clade B (ovalbumin), member 7; *DSG1*, Desmoglein1; *DSP*, Desmoplakin;*AAGAB*, Alpha- and gamma-adaptin-binding protein p34;*COL14A1*, Collagen XIV

## Conclusion

The identification of a novel homozygous nonsense variant in *SLURP1*, and a novel heterozygous nonsense variant in *DSG1*, as likely causes of PPK in the Pakistani families investigated alongside a review of previously reported variants adds to knowledge of the molecular causes of these conditions. Additionally, the data here provides important information regarding the nature, spectrum and molecular basis of PPK in Pakistan, enabling early clinical intervention, increased awareness regarding inherited disorders present in a community, and aiding diagnosis and counselling.

## Additional file

Additional file 1: **Table S1.** List of candidate pathogenic variants in *SLURP1* gene previously reported in association with Mal de Meleda. **Table S2.** List of candidate pathogenic variants in *DSG1* gene previously reported to be associated with Palmoplantar Keratoderma. (DOCX 66 kb)

## Data Availability

The patient’s non-sensitive datasets used and/or analyzed during the current study are available from the corresponding author on reasonable request.

## Abbreviations

ARS-B: Arylsulfatase B
*DSG1*: Desmoglein 1
HGMD: Human gene mutation data base
MDM: Mal de Meleda
OMIM: Online Mendelian inheritance in man
PPK: Palmoplantar Keratoderma
SAM: Sinobronchial allergic mycosis
*SERPIN7*: Serpin Peptidase Inhibitor, Clade B (Ovalbumin), Member 7
*SLURP1*: Secreted ly6/plaur domain-containing 1 gene
WES: Whole Exome Sequencing
