# Supplementary material for: Novel nonsense variants in SLURP1 and DSG1 cause palmoplantar keratoderma in Pakistani families
Source: BMC Med Genet. 2019 Aug 23;20:145. doi: 10.1186/s12881-019-0872-1 (PMC6708247; doi:10.1186/s12881-019-0872-1)
Supplement: Supplementary file 1 — Table S1. List of candidate pathogenic variants in SLURP1 gene previously reported in association with Mal de Meleda. Table S2. List of candidate pathogenic variants in DSG1 gene previously reported to be associated with Palmoplantar Keratoderma. (DOCX 66 kb) [file 12881_2019_872_MOESM1_ESM.docx]

**Supplementary File 1**

**Novel nonsense variants in *SLURP1* and *DSG1* cause palmoplantar keratoderma in Pakistani families**

Abida Akbar^1,2^, Claire Prince^2^, Chloe Payne^2^, James Fasham^2^, Wasim Ahmad^3^, Emma L Baple^2^, Andrew H Crosby^2^, Gaurav V Harlalka^2,4^,Asma Gul^1, *^

^1^Department of Biological Sciences, International Islamic University, H-10, Islamabad, 44000, Pakistan

^2^College of Medicine and Health, RILD Wellcome Wolfson Centre, University of Exeter, Royal Devon & Exeter NHS Foundation Trust, Barrack Road, Exeter, UK, EX2 5DW

^3^Department of Biochemistry, Faculty of Biological Sciences, Quaid-i-Azam University (QAU), Islamabad, Pakistan

^4^Rajarshi Shahu College of Pharmacy, Malvhir, Buldana, Maharashtra, Post code 443001, India

* Correspondence: gulasma@iiu.edu.pk

Dr Asma Gul,

Department of Biological Sciences, International Islamic University Islamabad, H-10, Islamabad, 44000, Pakistan

Tel: +92-51--9019837, Fax: +92-51- 9258012

| Variation type | Nucleotide change | Amino acid change | Reference |
| --- | --- | --- | --- |
| Missense | c.1A>C | p.Met1Leu | Eckl*et al*. [1] |
|  | c.2T>C | p.Met1Thr | Shah *et al*. [2] |
|  | c.43T>C | p.Trp15Arg | Eckl *et al*. [1]  Nellen *et al*. [3]  Zhao *et al*. [4] |
|  | c.211C>T | p.Arg71Cys | Radiono *et al*. [5] |
|  | c.212G>A | p.Arg71His | Favre *et al*. [6] |
|  | c.229T>C | p.Cys77Arg | Charfeddine *et al*. [7] |
|  | c.244C>T | p.Pro82Ser | Gruber *et al*. [8] |
|  | c.256G>A | p.Gly86Arg | Eckl*et al*. [1]  Chao *et al*. [9]  Chao *et al*. [10] |
|  | c.256G>C | p.Gly86Arg | Eckl *et al*. [1] |
|  | c.280T>A | p.Cys94Ser | Zhao *et al*. [4]  Nellen *et al*. [11] |
|  | c.293T>C | p.Leu98Pro | Yerebakan *et al*. [12] |
|  | c.296G>A | p.Cys99Tyr | Marrakchi *et al*. [13]  Bchetnia *et al*. [14] |
| Nonsense | **c.44G>A** | **p.Trp15*** | **Current study** |
|  | c.129C>A | p.Cys43* | Muslumanoglu *et al*. [15] |
|  | c.286C>T | p.Arg96* | Fischer *et al*. [16] |
| Frameshift | c.82delT | p.Cys28Alafs*5 | Fischer *et al*.[16] |
|  | c.275_277delTCT | p.Phe92del | Radiono *et al*.[5] |
| Splice-site | c.58+1G>A | Alteration of the WT donor site, predicted to affect splicing. | Wajid *et al*. [17] |
|  | c.58+1G>C |  | Sakabe *et al*. [18] |
|  | c.58+5G>T |  | Nellen *et al*. [19] |
|  | c.178+1G>A |  | Fischer *et al*. [16] |

**Supplementary table S1. List of candidate pathogenic variants in *SLURP1* gene previously reported in association with Mal de Meleda.**

| Variation type | Nucleotide change | Amino acid change | Reference |
| --- | --- | --- | --- |
| Nonsense | c.8G>A | p.Trp* | Lovgren *et al*. [20] |
|  | c.76C>T | p.Arg26* | Hunt *et al*. [21]  Lovgren *et al*. [20] |
|  | **c.133C>T** | **p.Arg45*** | **Current study** |
|  | c.395C>A | p.Ser132* | Kljuic *et al*. [22]  Nevet *et al*. [23] |
|  | c.430A>T | p.Arg144* | Zamiri *et al*. [24] |
|  | c.601C>T | p.Gln201* | Hershkovitz *et al*. [25] |
|  | c.655C>T | p.Arg219* | Hershkovitz *et al*. [25]  Fukaura *et al*. [26] |
|  | c.1095T>A | p.Tyr365* | Hunt *et al*. [21] |
|  | c.2659C>T | p.Arg887* | Has *et al*. [27] |
| Frameshift | c.121dupT | p.Trp41Leufs*10 | Milingou *et al*. [28] |
|  | c.277delG | p.Asp93Ilefs*19 | Scott *et al*. [29] |
|  | c.746dupT | p.Met249Ilefs*6 | Lovgren *et al*. [20] |
|  | c.811_812delCA | p.Gln271Valfs*20 | Danescu *et al*. [30] |
|  | c.1079dupC | p.Ile361Asnfs*6 | Hunt *et al*. [21] |
|  | c.1190delA | p.Asn397Ilefs*12 | Hunt *et al*. [21] |
|  | c.1560_1561delTT | p.Ser521Tyrfs*2 | Lovgren *et al*. [20] |
|  | c.1628delA | p.Asn543Metfs*19 | Hunt *et al*. [21] |
|  | c.1771_1784del14 | p.Asp591Phefs*10 | Lovgren *et al*. [20] |
|  | c.1861delG | p.Ala621Glnfs*3 | Samuelov *et al*. [32] |
|  | c.1892delG | p.Gly631Glufs*21 | Cheng *et al*. [33] |
|  | c.1931delA | p.Asp644Valfs*8 | Hershkovitz *et al*. [25] |
|  | c.2614delA | p.Ile872Serfs*10 | Schlipf *et al*. [34] |
| Splice-site | c.49-1G>A | Alteration of the WT acceptor site, predicted to affect splicing. | Samuelov *et al*. [32] |
|  | c.85-1G>A |  | Rickman *et al*. [35] |
|  | c.373-2A>G |  | Dua-Awereh *et al*. [31] |
|  | c.515C>T | Activation of an exonic cryptic donor site, predicted to affect splicing. | Dua-Awereh *et al*. [31] |
|  | c.517+5G>C | Alteration of the WT donor site, predicted to affect splicing. | Lovgren *et al*. [20] |
|  | c.1266-3C>G | Alteration of the WT acceptor site, predicted to affect splicing. | Dua-Awereh *et al*. [31] |
|  | c.1688-1G>T |  | Barber *et al*. [36] |
|  | c.1892-2A>C |  | Lee *et al*. [37] |
|  | c.2100+4A>G | Alteration of the WT donor site, predicted to affect splicing. | Danescu *et al*. [30] |

**Supplementary table S2. List of candidate pathogenic variants in *DSG1* gene previously reported to be associated with Palmoplantar Keratoderma.**

**REFERENCES**

[1] Eckl K, Stevens HP, Lestringant GG, Westenberger-Treumann M, Traupe H, Hinz B, Frossard PM, Stadler R, Leigh IM, Nürnberg P, Reis A. Mal de Meleda (MDM) caused by mutations in the gene for SLURP-1 in patients from Germany, Turkey, Palestine, andthe United Arab Emirates. Hum Genet. 2003;112(1):50-6.

[2] Shah K, Nasir A, Shahzad S, Khan S, Ahmad W. A novel homozygous mutation disrupting the initiation codon in the SLURP1 gene underlies Mal de Meleda in a consanguineous family. Clin Exp Dermatol. 2016;41(6):675-9.

[3] Nellen RG, Steijlen PM, Hennies HC, Fischer J, Munro CS, Jonkman MF, van Steensel MA, van Geel M. Haplotype analysis in western European patients with Mal de Meleda: founder effect for the W15R mutation in the SLURP1 gene. BrtJ Dermatol. 2013;168(6):1372-4.

[4] Zhao L, Vahlquist A, Vlrtanen M, Wennerstrand L, Lind LK, Lundstrom A, Pigg MH. Palmoplantar keratoderma of the Gamborg-Nielsen type is caused by mutations in the SLURP1 gene and represents a variant of Mal de Meleda.  Acta Derm Venereo. 2014;94(6):707-10.

[5] Radiono S, Pramono ZA, Oh GG, Surana U, Widiyani S, Danarti R. Identification of novel homozygous SLURP 1 mutation in a Javanese family with Mal de Meleda. IntJ Dermatol. 2017;56(11):1161-8.

[6] Favre B, Plantard L, Aeschbach L, Brakch N, Christen-Zaech S, de Viragh PA, Sergeant A, Huber M, Hohl D: SLURP1 is a late marker of epidermal differentiation and is absent in Mal de Meleda. Journal of Investtigative Dermatol. 2007;127(2):301-308.

[7] Charfeddine C, Mokni M, Ben Mousli R, Elkares R, Bouchlaka C, Boubaker S, Ghedamsi S, Baccouche D, Ben Osman A, Dellagi K, Abdelhak S. A novel missense mutation in the gene encoding SLURP‐1 in patients with Mal de Meleda from northern Tunisia. Brt J Dermatol. 2003;149(6):1108-15.

[8] Gruber R, Hennies HC, Romani N, Schmuth M. A novel homozygous missense mutation in SLURP1 causing Mal de Meleda with an atypical phenotype. Arch dermatol. 2011;147(6):748-50.

[9] Chao SC, Lai FJ, Yang MH, Lee JY. Mal de Meleda in a taiwanese. J Formos Med Assoc. 2005;104(4):276-8.

[10] Chao SC, Huang CY, Lai FJ, Yang MH, Chao SC. Pseudodominant inheritance with the G86R mutation in the ARS gene in Mal de Meleda. Int J Dermatol. 2006;45(12):1456-8.

[11] Nellen RG, Steijlen PM, van Geel M, van Steensel MA, Zhao L, Vahlquist A, Virtanen M, Wennerstrand L, Lind LK, Lundström A, Pigg MH. Comment on Zhao et al.“Palmoplantar Keratoderma of the Gamborg-Nielsen Type is Caused by Mutations in the SLURP1 Gene and Represents a Variant of Mal de Meleda” Reply to Nellen et al's Comment on the Classification of Clinical/genetic Variants of Mal de Meleda. Acta dermato-venereologica. 2015;95(8):1034-5.

[12] Yerebakan O, Hu G, Yilmaz E, Celebi JT: A novel mutation in the ARS (component B) gene encoding SLURP-1 in a family with Mal de Meleda. Clin Exp Dermatol. 2003; 28(5):542-544.

[13] Marrakchi S, Zahaf A, Turki H, Audebert S, Bouadjar B, Has C, Lefèvre C, Munro C, Cure S, Jobard F, Morlot S. Novel mutations in the gene encoding secreted lymphocyte antigen-6/urokinase-type plasminogen activator receptor-related protein-1 (SLURP-1) and description of five ancestral haplotypes in patients with Mal de Meleda. J Invest Dermatol. 2003;120(3):351-5.

[14] Bchetnia M, Merdassi A, Charfeddine C, Mgaieth F, Kassar S, Ouechtati F, Chouchene I, Boussen H, Mokni M, Osman AD, Boubaker MS. Coexistence of Mal de Meleda and congenital cataract in a consanguineous Tunisian family: two case reports. J Med Case Rep. 2010;4(1):108.

[15] Muslumanoglu MH, Saracoglu N, Cilingir O, Basmaci T, Urer S, Sabuncu I, Demir S, Bademci G, Artan S. A novel mutation in the ARS (component B) gene encoding SLURP‐1 in a Turkish family with Mal de Meleda. Brt J Dermatol. 2006;155(2):467-9.

[16] Fischer J, Bouadjar B, Heilig R, Huber M, Lefèvre C, Jobard F, Macari F, Bakija-Konsuo A, Ait-Belkacem F, Weissenbach J, Lathrop M. Mutations in the gene encoding SLURP-1 in Mal de Meleda. Hum Mol Genet. 2001;10(8):875-80.

[17] Wajid M, Kurban M, Shimomura Y, Christiano AM. Mutations in the SLURP-1 gene underlie Mal de Meleda in three Pakistani families. J dermatol sci. 2009;56(1):27-32.

[18] Sakabe JI, Kabashima‐Kubo R, Kubo A, Sasaki T, Tokura Y. AJ apanese case of M al de M eleda with SLURP 1 mutation. J dermatol. 2014;41(8):764-5.

[19] Nellen RG, Claessens T, Subramaniam R, Betkerur J, Prashanth A, Steijlen PM, van Geel M. A novel mutation in SLURP1 in patients with Mal de Meleda from the Indian subcontinent. J dermatol sci. 2015;80(1):76-8.

[20] Lovgren ML, McAleer MA, Irvine AD, Wilson NJ, Tavadia S, Schwartz ME, Cole C, Sandilands A, Smith FJ, Zamiri M. Mutations in desmoglein 1 cause diverse inherited palmoplantar keratoderma phenotypes: implications for genetic screening. Brt J Dermatol. 2017;176(5):1345-50.

[21] Hunt D, Rickman L, Whittock N, Eady R, Šimrak D, Dopping-Hepenstal P, Stevens H, Armstrong D, Hennies H, KuÈster W, Hughes A. Spectrum of dominant mutations in the desmosomal cadherin desmoglein 1, causing the skin disease striate palmoplantar keratoderma. Eur J Hum Genet. 2001;9(3):197.

[22] Kljuic A, Gilead L, Martinez‐Mir A, Frank J, Christiano AM, Zlotogorski A. A nonsense mutation in the desmoglein 1 gene underlies striate keratoderma. Exp Dermatol. 2003;12(4):523-7.

[23] Nevet MJ, Indelman M, Bergman R. Acantholysis in Striate Keratoderma as a Clue to the Diagnosis of a Genetic Abnormality. Am J Dermatol. 2015;37(10):804-5

[24] Zamiri M, Smith FJ, Campbell LE, Tetley L, Eady RA, Hodgins MB, McLean WH, Munro CS. Mutation in DSG1 causing autosomal dominant striate palmoplantar keratoderma. Brt J Dermatol. 2009;161(3):692-4.

[25] Hershkovitz D, Lugassy J, Indelman M, Bergman R, Sprecher E. Novel mutations in DSG1 causing striate palmoplantar keratoderma. Clinical and Experimental Dermatology: Exp Dermatol. 2009;34(2):224-8.

[26] Fukaura R, Takeichi T, Okuno Y, Kojima D, Kono M, Sugiura K, Suga Y, Akiyama M. Striate palmoplantar keratoderma showing transgrediens in a patient harbouring heterozygous nonsense mutations in both DSG1 and SERPINB7.Acta Derm Venereo. 2017;97(3):399-401.

[27] Has C, Jakob T, He Y, Kiritsi D, Hausser I, Bruckner‐Tuderman L. Loss of desmoglein 1 associated with palmoplantar keratoderma, dermatitis and multiple allergies. Brt J Dermatol. 2015;172(1):257-61.

[28] Milingou M, Wood P, Masouye I, McLean WH, Borradori L. Focal palmoplantar keratoderma caused by an autosomal dominant inherited mutation in the desmoglein 1 gene. Dermatol. 2006;212(2):117-22.

[29] Scott CA, Plagnol V, Nitoiu D, Bland PJ, Blaydon DC, Chronnell CM, Poon DS, Bourn D, Gárdos L, Császár A, Tihanyi M. Targeted sequence capture and high-throughput sequencing in the molecular diagnosis of ichthyosis and other skin diseases. JInvest Dermatol. 2013;133(2):573.

[30] Dănescu S, Leppert J, Cosgarea R, Zurac S, Pop S, Baican A, Has C. Compound heterozygosity for dominant and recessive DSG 1 mutations in a patient with atypical SAM syndrome (severe dermatitis, multiple allergies, metabolic wasting). J Eur Acad Dermatol Venereol. 2017;31(3):144-6.

[31] Dua-Awereh MB, Shimomura Y, Kraemer L, Wajid M, Christiano AM. Mutations in the desmoglein 1 gene in five Pakistani families with striate palmoplantar keratoderma. J Dermatol Sci. 2009;53(3):192-7.

[32] Samuelov L, Sarig O, Harmon RM, Rapaport D, Ishida-Yamamoto A, Isakov O, Koetsier JL, Gat A, Goldberg I, Bergman R, Spiegel R. Desmoglein 1 deficiency results in severe dermatitis, multiple allergies and metabolic wasting. Nat Genet. 2013;45(10):1244.

[33] Cheng R, Yan M, Ni C, Zhang J, Li M, Yao Z. Report of Chinese family with severe dermatitis, multiple allergies and metabolic wasting syndrome caused by novel homozygous desmoglein‐1 gene mutation. J Dermatol. 2016;43(10):1201-4.

[34] Schlipf NA, Vahlquist A, Teigen N, Virtanen M, Dragomir A, Fismen S, Barenboim M, Manke T, Rösler B, Zimmer A, Fischer J. Whole-exome sequencing identifies novel autosomal recessive DSG1 mutations associated with mild SAM syndrome. Brt J Dermatol. 2016;174(2):444.

[35] Rickman L, Šimrak D, Stevens HP, Hunt DM, King IA, Bryant SP, Eady RA, Leigh IM, Arnemann J, Magee AI, Kelsell DP. N-terminal deletion in a desmosomal cadherin causes the autosomal dominant skin disease striate palmoplantar keratoderma. Hum Mol Genet. 1999;8(6):971-6.

[36] Barber AG, Wajid M, Columbo M, Lubetkin J, Christiano AM. Striate palmoplantar keratoderma resulting from a frameshift mutation in the desmoglein 1 gene. J Dermatol Sci. 2007;45(3):161-6.

[37] Lee JY, Farag A, Tawdy A, Liu L, Michael M, Rashidghamat E, Aristodemou S, Hsu CK, Simpson MA, Parsons M, McGrath JA. Homozygous acceptor splice site mutation in DSG1 disrupts plakoglobin localization and results in keratoderma and skin fragility. J Dermatol Sci. 2018;89(2):198-201.
